# The association between compassion fatigue and nurses’ creativity: the mediating role of ego depletion

**DOI:** 10.3389/fpsyg.2026.1748888

**Published:** 2026-07-15

**Authors:** Bo Wang, Zhaoxia Wang, Guilian Wang, Heqian Lv, Hui Qi, Jie Li

**Affiliations:** 1 Department of Neurology, First Hospital of Shanxi Medical University, Taiyuan, Shanxi, China; 2 Emergency Department, Second Hospital of Shanxi Medical University, Taiyuan, Shanxi, China; 3 College of Nursing, Changzhi Medical College, Changzhi, Shanxi, China; 4 Shanxi Key Laboratory of Otorhinolaryngology-Head and Neck Cancer, First Hospital of Shanxi Medical University, Taiyuan, Shanxi, China

**Keywords:** compassion fatigue, creativity, ego depletion, the mediating role, nurse

## Abstract

**Introduction:**

Compassion fatigue is a prevalent occupational hazard among nurses that may undermine their creative performance, yet the underlying mechanism remains underexplored. This study aimed to examine the mediating role of ego depletion in the relationship between compassion fatigue and creativity among clinical nurses.

**Methods:**

A cross-sectional design was adopted. A total of 1,055 nurses were recruited from four tertiary general hospitals in China between September 2024 and January 2025. Data were collected using a general information questionnaire, the Self‑Regulatory Fatigue Scale, the Professional Quality of Life Scale Version 5, and the Employee Creativity Scale. Structural equation modeling was applied to assess the mediating effect of ego depletion. Ultimately, 950 nurses completed the survey.

**Results:**

The results showed that: (1) Compassion fatigue among nurses was at a relatively high level, while ego depletion and creativity were at moderate levels. (2) Creativity was significantly and negatively correlated with both compassion fatigue and ego depletion, as well as with all subscale scores. (3) Compassion fatigue had a direct negative association with creativity. Mediation analysis further revealed that ego depletion partially mediated this relationship, with a significant indirect effect.

**Conclusion:**

Ego depletion serves as a partial mediator in the link between compassion fatigue and creativity. These findings suggest that alleviating compassion fatigue and preventing ego depletion may help preserve nurses’ professional vitality and sustain their creative performance. Nursing administrators should prioritise interventions targeting both factors.

## 1 Introduction

Compassion fatigue refers to a state of physical and emotional exhaustion and imbalance experienced by helpers due to prolonged exposure to empathy-related stress (Figley, 2002). According to Stamm (2010), compassion fatigue is a multidimensional construct comprising two core subcomponents: burnout and secondary traumatic stress. Secondary traumatic stress is caused by the care providers exposure to the suffering of others who have or are experiencing stressful events. Burnout refers to the psychological experience resulting from a decline in self-efficacy among care providers due to increased workload demands and heightened perceived stress. It is important to distinguish compassion fatigue from the related but distinct concept of empathy fatigue. Empathy fatigue is a state of exhaustion resulting from the repeated cognitive and emotional effort involved in adopting another person’s perspective and sharing their emotions; it does not necessarily require exposure to a full account of a traumatic experience (Stebnicki, 2007; Gleichgerrcht and Decety, 2013). Despite this distinction, the two constructs share critical similarities: both are occupational hazards for helping professionals, both involve progressive exhaustion of emotional and cognitive resources, and both lead to reduced caregiving responsiveness and increased personal distress (Cocker and Joss, 2016). This study focuses on compassion fatigue because it is a well-established construct with a validated measurement tool (the Professional Quality of Life Scale version 5, ProQOL-5) that is widely used in nursing populations.

Clinical nurses are exposed to a high-intensity, high-stress work environment, requiring them to directly, frequently, and extensively interact with patients in distress. While performing intensive diagnostic and therapeutic procedures to ensure medical accuracy, they must also continuously perceive and respond to patients’ potential emotional needs. These factors collectively place nurses at high risk for Compassion fatigue (Tian et al., 2018). The occupational health challenges faced by nurses constitute a significant global concern. Nurse burnout is highly prevalent worldwide, with estimated prevalence rates ranging from 30 to 60%. Among its dimensions, emotional exhaustion affects approximately 33% of nurses, depersonalization about 25%, and these conditions are frequently accompanied by anxiety or depression, with prevalence rates ranging from 23 to 61% (Fekih-Romdhane et al., 2025; Ge et al., 2023; Getie et al., 2025; Norful et al., 2024; Squires et al., 2025). In the Asian context, the rapid expansion of healthcare systems, an excessively high patient-to-nurse ratio, culturally ingrained expectations of emotional restraint, and an imbalance between work demands and rewards have collectively contributed to an increase in the incidence of stress-related illnesses. Specifically in China, the nursing workforce density was 4.32 per 1,000 population by the end of 2025 (National Health Commission of China, 2026), substantially lower than the OECD average of 9.2 practicing nurses per 1,000 population in 2023 (OECD, 2025). Consequently, Chinese nurses face prolonged overtime shifts, heightened workplace violence, and intense performance pressure. Empirical research indicates that over 43% of Chinese nurses report high levels of job-related stress, approximately 35% experience clinically significant psychological distress, and nearly 50% exhibit reduced professional efficacy, which is a core symptom of burnout. Moreover, more than 54% of nurses fall into moderate to severe burnout categories, with 44.6% classified as “moderate burnout-exhausted” and 10.0% as “severe burnout-dysfunctional” (Ren et al., 2023; Yang et al., 2025; Wu et al., 2021; Li et al., 2026). This landscape of pervasive occupational distress underscores the urgency of investigating stress-related psychological constructs like compassion fatigue within the Chinese nursing population. Research indicates that empathy fatigue leads to gradual mental, physical, and emotional exhaustion among nurses (Cao et al., 2025), not only affecting their job satisfaction and physical and mental health (Wang et al., 2024), but also diminishing their attention to patients, reducing team cohesion, and lowering work motivation (Yuan et al., 2019; Zhong et al., 2025). Based on these negative impacts, this study hypothesizes that empathy fatigue will exert a detrimental effect on nurses’ creativity.

Creativity is the process by which individuals generate novel ideas or technologies in practical work, modify previously used procedures, and thereby achieve efficient performance (Hoever et al., 2012). With technological advancements and societal transformations, the healthcare system must rely on creativity to effectively address emerging needs and complex clinical scenarios (Denhardt et al., 2018). It is projected that nearly 70% of the nursing workforce will operate in highly unpredictable environments. Nurses need to develop interpersonal, higher-order cognitive, and system-level competencies, alongside complementary skills in personal and customer services, decision making, technology utilization, creativity, and scientific methodologies (Notarnicola et al., 2025). As core members of the healthcare team, nurses frequently encounter unexpected situations that deviate from standard operating procedures, placing heightened demands on their professional competence, creative thinking, and ability to respond promptly (Institute of Medicine [IOM], 2011; Dresser et al., 2023). In clinical settings, creativity manifests specifically as nurses’ ability to generate novel ideas, resolve complex problems, and make effective decisions (Bunkers, 2011). This capability enables nurses to think outside the box, optimize nursing processes, develop more effective patient education tools, or refine resource allocation strategies, thereby directly enhancing the quality of care and patient outcomes (Isfahani et al., 2015; Klawunn et al., 2023). Empirical studies further indicate that nurses’ creativity are significantly associated with reduced medical errors and enhanced patient safety, particularly in complex case management where creative problem-solving effectively mitigates clinical risks (Malik et al., 2016; van Houwelingen et al., 2023). Therefore, thoroughly exploring and supporting the development of nurses’ creativity is a key strategic direction for enhancing the overall quality of healthcare services in the future.

This study analyzes the phenomenon based on resource conservation theory (Hobfoll, 1989). The theory posits that individuals respond to threats or losses of existing resources by conserving valuable resources, meaning that the impact of resource loss is far more significant than the impact of resource acquisition. Long-term compassion fatigue gradually depletes nurses’ limited energy, time, and resources, potentially leading to ego depletion (Cui et al., 2023). Ego depletion measures the decline in an individual’s volitional capacity or willingness to exert self-control during such efforts, leading to reduced behavioral self-management and diminished self-management beliefs (Baumeister and Boden, 1998). In nursing practice, ego depletion may lead to deficiencies in self-management behaviors, inadequate emotional self-regulation, and diminished self-management beliefs (Roczniewska, 2021), which can subsequently reduce nursing effectiveness, compromise care quality, and even jeopardize patient safety (Guo et al., 2025). When nurses are in a state of resource conservation, their execution and willpower diminish, making it difficult to maintain mental alertness and emotional focus. Consequently, they struggle to generate rational, proactive, and innovative behaviors (Kim and Beehr, 2022). Although existing research has identified numerous factors influencing nurses’ compassion fatigue and its negative impact on work behaviors, critical research gaps remain. First, while resource conservation theory provides a compelling framework for understanding how emotional labor leads to resource loss, no empirical study has directly tested whether ego depletion serves as the psychological mechanism linking compassion fatigue to impaired work outcomes in nurses. Specifically, the pathway from compassion fatigue to ego depletion has been theorized but not empirically validated in clinical nursing populations. Second, although ego depletion has been shown to impair creativity in domains such as education and organizational management (Zhang et al., 2024; Bollimbala et al., 2021), the mediating role of ego depletion in the compassion fatigue–creativity relationship remains completely unexplored in healthcare settings. Third, existing studies have examined compassion fatigue and creativity as separate phenomena, but no research has integrated these constructs within a single, theory-driven model that specifies ego depletion as the intervening variable. Therefore, the present study addresses these gaps by testing a mediation model grounded in resource conservation theory.

Based on the theoretical arguments and identified research gaps, we propose the following hypotheses:

*Hypothesis* 1 (H1): Compassion fatigue is negatively associated with creativity among clinical nurses.

*Hypothesis* 2 (H2): Ego depletion is negatively associated with nurses’ creativity.

*Hypothesis* 3 (H3): Ego depletion mediates the negative relationship between compassion fatigue and nurses’ creativity.

## 2 Methods

### 2.1 Participants and procedures

This study employed a cross-sectional design. The reporting of this cross-sectional study follows the STROBE (Strengthening the Reporting of Observational Studies in Epidemiology) checklist. Convenience sampling was used to select clinical nurses from four tertiary-level hospitals in Shanxi Province, China as research subjects between September 2024 and January 2025. They are public general hospitals with bed capacities ranging from 1,500 to 3,000. These hospitals were selected based on geographic accessibility, established collaboration between our research team and their nursing departments, and their representative patient volume and nurse workload in the region. Inclusion criteria: ① Registered nurses with more than 1 year of clinical nursing experience; This criterion was adopted to ensure that participants had sustained, repeated exposure to clinical stressors, as compassion fatigue is conceptualized as a cumulative process (Figley, 2002); newly hired nurses (less than 1 year) are often still in an adaptation phase and may exhibit systematically different patterns of compassion fatigue (Rudman and Gustavsson, 2012). ② Informed consent and voluntary participation. Exclusion criteria: ① Nurses who do not come into direct contact with patients, such as nurses in the sterilization supply room or medication preparation center; nurses on rotation or training; ② Nurses who were not on duty during the investigation period. ③ Experienced a major life event within the past 3 months (such as divorce, death or serious illness of spouse, child, or parent). According to the structural equation modeling requirements, the sample size should be no less than 200 (Wu, 2009). Considering that there may be 20% invalid samples, the minimum sample size is 250.

The four participating hospitals do not have formal employee assistance programs (EAPs) or structured psychological counseling services specifically for nurses. Mental health support is limited to informal peer discussions and occasional hospital-wide lectures on stress management. No regular debriefing sessions or clinical supervision are provided. This lack of organizational support may exacerbate the resource depletion process described in our findings.

The study was conducted in accordance with the 1964 Helsinki Declaration and its subsequent amendments. This study has been approved by the Ethics Committee of the First Hospital of Shanxi Medical University (KYLL-2024-278). All participants have provided informed consent and voluntarily participated in this survey.

### 2.2 Measures

Demographic Information. The demographic information questionnaire encompassed several demographic and professional variables. Designed by the researchers themselves, including age, years of work experience, gender, marital status, educational background, professional title, employment status, and department of work.

Ego Depletion. The Self-Regulated Fatigue Scale (SRF-S) was used to assess ego depletion. The SRF-S was originally developed by Nes et al. (2013) to evaluate an individual’s level of ego depletion while differentiating it from physical fatigue. Wang et al. (2015) adapted the SRF-S into Chinese. The revised Chinese version comprises three domains with 16 items: six assessing cognitive fatigue, five evaluating behavioral control, and five measuring emotional control. Items are rated on a 5-point Likert scale, yielding a total score ranging from 16 to 80, with higher scores indicating greater ego depletion. The Chinese version demonstrates acceptable psychometric properties, with a Cronbach’s *α* coefficient of 0.84 and a test–retest reliability of 0.73 (Wang et al., 2015), indicating good reliability and validity. This instrument has been widely applied across diverse populations and is currently the most commonly used tool for assessing ego depletion.

Compassion Fatigue. The assessment of compassion fatigue was conducted using the Professional Quality of Life Scale, Version 5 (ProQOL-5). This scale was originally developed by Stamm (2010) and subsequently translated into Chinese by Chen and Wang (2013). It comprises three domains: compassion satisfaction, job burnout, and secondary traumatic stress, with a total of 10 items allocated to each domain, totaling 30 items. Responses are rated on a 5-point Likert scale ranging from “never” to “always,” corresponding to scores from 1 to 5. The maximum total score for each domain is 50. Higher scores in compassion satisfaction reflect greater personal fulfillment, whereas higher scores in the job burnout and secondary traumatic stress domains indicate more severe levels of compassion fatigue. Based on the two-dimensional conceptualization of compassion fatigue, researchers commonly define compassion fatigue scores as the sum of the scores from the two negative domains (job burnout and secondary traumatic stress) of this instrument (Li, 2018).

Creativity. Creativity was assessed using the Employees’ Creativity Scale, originally developed by George and Zhou (2001). Guo (2016) later simplified and adapted the scale in 2011, reducing the number of items from 13 to 10. The revised 10-item scale has been empirically validated. A 5-point Likert scale was used, with response options ranging from “Strongly Disagree” to “Strongly Agree,” allowing participants to respond according to their actual experiences and subjective perceptions. Responses were scored from 1 to 5, with higher scores reflecting stronger agreement. The overall scale demonstrated a Cronbach’s *α* coefficient of 0.945.

### 2.3 Data collection methods

After obtaining approval from the nursing department of the target hospital, the researcher personally contacted the head nurse of the department, explained the purpose and significance of the survey, and secured their cooperation. Train department head nurses on the survey completion method and key considerations, emphasizing the questionnaire’s anonymity and confidentiality. Instruct them to complete it independently and clarify inclusion and exclusion criteria. This study adheres to the principle of voluntary participation. Clear instructions are provided on the questionnaire cover page and at the beginning of each section. All participants complete informed consent by clicking the “I Agree” option below the instructions above. The head nurses shared a hyperlink and a QR code of the electronic questionnaire (created using Wenjuanxing) in their department’s WeChat group. A standardized text message was provided to the head nurses to copy and send, which included: (1) a brief explanation of the study purpose, (2) assurance of anonymity and confidentiality, (3) the voluntary nature of participation, (4) a request to complete the questionnaire independently without discussion, (5) the same IP address can only be entered once, (6) a 72-h deadline for submission. One reminder message was sent by the head nurse after 48 h to non-respondents, and (7) the electronic questionnaire enforced a ‘forced response’ option, requiring participants to answer all items before submission. After the survey concluded, three researchers reviewed each collected questionnaire individually, excluding those with answers exhibiting obvious patterns or uniformity, as well as those completed in under 300 s. The three reviewers were members of the research team: one master’s student in nursing and two clinical nurses with master’s degrees in nursing who served as research assistants for this study. They had access to the raw data only after collection was complete and did not have the ability to modify any individual responses. A pilot test was conducted with two clinical nurses who were not included in the final sample. They completed the electronic questionnaire and confirmed that the instructions were clear, the items were understandable, and the completion time was approximately 8–10 min. No revisions were needed.

### 2.4 Statistical methods

Data analysis was conducted using SPSS version 26.0 statistical software. For quantitative data that did not follow a normal distribution, median and interquartile range were used for descriptive purposes. Categorical data were described using frequencies and proportions. Pearson correlation analysis was performed to assess associations among variables. Structural equation modeling was carried out using AMOS 24.0 software. The mediating effect was evaluated using the Bootstrap method, with 5,000 resamples to estimate 95% confidence intervals.

## 3 Result

### 3.1 General information

As detailed in section 2.3, a total of 1,055 questionnaires were distributed and 950 valid responses were obtained (effective response rate 90%). The demographic characteristics of the 950 nurses are as follows: the 950 nurses ranged in age from 24 to 57 years old, with a median age of 32; their years of service spanned 1 to 35 years, with a median of 9 years; 72 were male and 878 were female; 794 were married, while 156 were unmarried or divorced; 36 held master’s degrees or higher, 900 held bachelor’s degrees, and 14 held junior college degrees or lower; 676 held the title of Senior Nurse or higher, while 274 held the title of Nurse or lower. Employment status: 173 were permanent staff, and 777 were contract employees. Departments: Internal Medicine (178), Surgery (164), Intensive Care Unit (ICU; 120), Emergency Department (124), Obstetrics and Pediatrics (120), Outpatient Department (128), Operating Room (116). General characteristics of the survey participants are shown in Table 1.

**Table 1:** General information of surveyed patients (*N* = 950).

Characteristic	Category	n (%)	Characteristic	Category	n (%)
Sex	Male	72 (7.6)	Type of employment	Permanent	173 (18.2)
	Female	878 (92.4)		Contract and other	777 (81.8)
Marital status	Married	794 (83.6)	Department	Internal Medicine	178 (18.7)
	Unmarried	156 (16.4)		Surgery	164 (17.3)
Education	Junior college	14 (1.5)		ICU	120 (12.6)
	Bachelor’s degree	900 (94.7)		Emergency Department	124 (13.1)
	Masters and above	36 (3.8)		Obstetrics and Pediatrics	120 (12.6)
Positional titles	Junior	274 (28.8)		Outpatient Department	128 (13.5)
	Intermediate	654 (68.8)		Operating Room	116 (12.2)
	Vice-senior or above	22 (2.3)			

### 3.2 The scores of ego depletion, compassion fatigue and creativity

Among 950 clinical nurses, the average total score on the ego depletion was 45.0(39.0, 50.0) points, with scores of 17.4 (15.0, 14.0) points on the cognitive control dimension, 13.7 (11.0, 16.0) points on the behavioral control dimension, and 13.8 (12.0, 15.0) points on the emotional control dimension. The average total score for the compassion fatigue was 69 (62.0, 75.0) points, of which 32.9 (30.0, 36.0) was for job burnout dimension, and the score for the secondary traumatic stress dimension was 36.1 (32.0, 40.0). The average total score for employees creativity was 32.3 (28.0, 37.0). For detailed scores on each scale, see Table 2.

**Table 2:** The scores of ego depletion, compassion fatigue and creativity [*n* = 950, M(P25, P75)].

Variables	Entry score	Total score
SRF-S	15.0 (13.0, 16.7)	45.0 (39.0, 50.0)
Cognitive dimension	2.9 (2.5, 3.2)	17.4 (15.0, 19.0)
Behavioral control dimension	2.7 (2.2, 3.2)	13.7 (11.0, 16.0)
Emotional control dimension	2.8 (2.4, 3.0)	13.8 (12.0, 15.0)
ProQOL-5	34.5 (31.0, 37.5)	69 (62.0, 75.0)
Job burnout	3.2 (3.0, 3.6)	32.9 (30.0, 36.0)
Secondary traumatic stress	3.6 (3.2, 4.0)	36.1 (32.0, 40.0)
Employees creativity	3.2 (2.8, 3.7)	32.3 (28.0, 37.0)

SRF-S, ego depletion; ProQOL-5, compassion fatigue; Employees Creativity, creativity.

### 3.3 The correlation analysis among ego depletion, compassion fatigue and creativity

Table 3 shows the results of the correlation analysis between ego depletion, compassion fatigue and creativity. There was a negative correlation between compassion fatigue and creativity (*r* = −0.698, *p* < 0.001). There was a positive correlation between ego depletion and compassion fatigue (*r* = 0.737, *p* < 0.01). There was a negative correlation between ego depletion and creativity (*r* = −0.656, *p* < 0.01).

**Table 3:** The correlation analysis between ego depletion, compassion fatigue and creativity(r value).

Variables	ProQOL-5	SRF-S	Employees creativity
ProQOL-5	1.000	-	-
Job burnout	0.915	0.731	−0.675
Secondary traumatic stress	0.926	0.632	−0.616
SRF-S	0.737	1.000	-
Cognitive dimension	0.581	0.781	−0.540
Behavioral control dimension	0.680	0.893	−0.590
Emotional control dimension	0.634	0.901	−0.576
Employees Creativity	−0.698	−0.656	1.000

ProQOL-5, compassion fatigue; SRF-S, ego depletion; Employees Creativity, creativity.

### 3.4 Mediation analysis between nurses’ compassion fatigue, ego depletion and creativity

**Figure 1:**
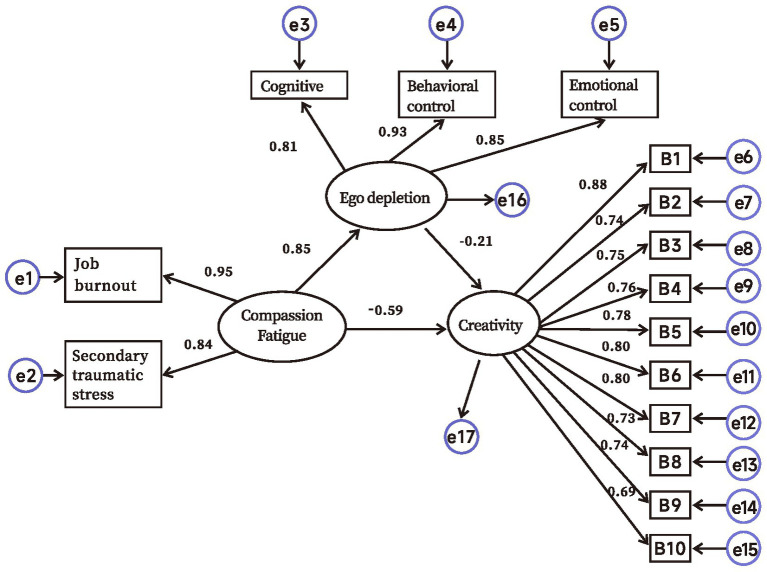
A mediated model of ego depletion on nurse compassion fatigue and nurse creativity.

Using AMOS 24.0 software, a structural equation model was constructed based on resource conservation theory (Hobfoll, 1989), with compassion fatigue as the independent variable, creativity as the dependent variable, and ego depletion as the mediating variable. The model was refined based on revised indicators and specific circumstances, ultimately yielding the final model depicted in Figure 1.

Model fit indices: Chi-square/degrees of freedom (χ^2^/df) = 4.376 < 5.000; Root Mean Square Error of Approximation (RMSEA) = 0.060 < 0.080. A smaller χ^2^/df value indicates better model fit, while a smaller RMSEA value signifies superior model adequacy. Goodness-of-Fit Index (GFI) = 0.960 > 0.900, Adjusted Goodness-of-Fit Index (AGFI) = 0.927 > 0.900, Comparative Fit Index (CFI) = 0.980 > 0.900, and Tolerance-to-Liberty Index (TLI) = 0.968 > 0.900. All indices fall within acceptable ranges, indicating good model fit.

AMOS analysis results indicate that nurses’ compassion fatigue and ego depletion are negatively associated with their creativity, as shown in Table 4. To better assess the significance of the mediating effect, the Bootstrap method was employed to resample the original data 5,000 times. A 95% confidence interval (CI) was calculated for the mediating effect, with the interval excluding zero. The results indicate that nurses’ ego depletion partially mediates the effect of compassion fatigue on nurses’ creativity. The total effect of nurses’ compassion fatigue on creativity was −0.769 (*p* < 0.001, 95%CI: −0.824 to −0.710), with a direct effect of −0.593 (*p* < 0.001, 95%CI: −0.785 to −0.380), with a mediation effect of −0.176 (*p* < 0.001, 95%CI: −0.360 to −0.027), accounting for 22.89% of the total effect.

**Table 4:** Effect of nurse compassion fatigue and ego depletion on creativity.

Path	Standardized	Unstandardized	t	*p*
Compassion fatigue→creativity	−0.59	−0.11	−10.750	<0.001
Compassion fatigue→ego depletion	0.85	0.49	23.793	<0.001
Ego depletion→creativity	−0.21	−0.06	−3.886	<0.001

### 3.5 Common method bias test

Harman’s single-factor test was used to examine common method bias across all variables. The results showed that a total of 4 factors with eigenvalues greater than 1 were extracted. The first factor accounted for 31.32% of the variance, which is below the critical threshold of 40% (Zhou and Long, 2004), indicating no significant common method bias in this study.

## 4 Discussion

This study is the first to simultaneously examine the levels of compassion fatigue, ego depletion, and creativity among Chinese clinical nurses, as well as the mediating role of ego depletion in the relationship between compassion fatigue and creativity. Our findings reveal that compassion fatigue is relatively high (median = 34.5), while ego depletion (median = 45.0) and creativity (median = 32.3) are at moderate levels. More importantly, the negative direct effect of compassion fatigue on creativity (*β* = −0.593, *p* < 0.001) was partially mediated by ego depletion (indirect effect *β* = −0.176, 95% bootstrap CI: −0.360 to −0.027, accounting for 22.89% of the total effect). These results provide new insights into the emotional and cognitive resource mechanisms underlying nurses’ innovative behavior, while also highlighting context-specific patterns that differ from those observed in Western healthcare systems.

In this study, the total compassion fatigue score among clinical nurses was 34.5 (31.00, 37.5), indicating a relatively high level. This finding aligns with reports from China (Liu et al., 2023) and Iran (Mottaghi et al., 2020), suggesting that nurses in collectivistic, high-workload Asian healthcare settings are particularly susceptible to compassion fatigue. However, cross-country comparisons reveal important differences. For instance, a US study among nurses in Texas (Crabtree-Nelson et al., 2022) reported low compassion fatigue (mean = 21.4), whereas our study found higher compassion fatigue. This discrepancy likely stems from the US nurses working fewer hours per week (many ≤35 h), and more favorable nurse-to-patient ratios, structured psychological supervision, and a stronger culture of emotional boundary-setting. Chinese nurses in our sample were more affected by chronic workload, appeasement management, and lack of administrative support. These discrepancies underscore the need to consider national healthcare policies and cultural norms regarding emotional labor when interpreting compassion fatigue levels.

The high compassion fatigue in our sample can be explained by several factors. The increasing complexity of healthcare, intense interpersonal interactions (Missouridou, 2017), excessive psychological demands, and workplace stress are exacerbated by severe nursing shortages, mandatory overtime, inadequate rest after night shifts, and a hierarchical management style that often dismisses nurses’ emotional needs (Maillet and Read, 2021; Coetzee and Klopper, 2010). Moreover, in the Chinese cultural context, nurses are socialized to suppress personal distress and maintain a compassionate facade (“ren” – endurance), which paradoxically increases compassion fatigue over time.

The results of this study indicate that nurses’ ego depletion scores averaged 45.0 (39.0, 50.0), reflecting a moderately high level. This finding is not only consistent with previous studies conducted in China (Zhang et al., 2021; Zhou et al., 2025), but also aligns with the levels of related constructs reported among international nurse samples. For instance, comparable levels of self-regulatory fatigue have been documented in German long-term care nurses (Schmidt, 2010), and decision fatigue scores among U. S. clinical nurses (Pignatiello et al., 2022) as well as Korean registered nurses (Hur and Hickman, 2024) similarly indicate a significant depletion of self-regulatory resources. Although the specific measures differ, the convergence of evidence across diverse cultural contexts suggests that a moderately high level of resource depletion is a prevalent phenomenon among nurses globally. According to Baumeister’s energy model (Baumeister, 2003), nurses continuously expend limited self-control resources or energy not only in addressing clinical patient issues but also in persistently confronting and resolving numerous other workplace challenges. This depletion adversely affects subsequent self-control behaviors, such as diminished or even failed self-management capabilities. Resource conservation theory (Hobfoll, 1989) indicates that when nurses facing various stresses fail to recognize available resources around them, the pressure and negative emotions they endure will continuously intensify. This leads to issues of ego depletion, such as professional apathy, loss of occupational interest, negative work attitudes, and questioning the meaning of the nursing profession (Zhang et al., 2021). When individuals experience ego depletion, they become more susceptible to negative influences. Consequently, issues arising from ego depletion not only reduce nurses’ work enthusiasm and efficiency, compromising the quality of nursing care (Labrague et al., 2025), but also significantly impair their creative performance (Weiss et al., 2022).

The results of this study indicate that the total score for nurses’ creativity was 32.3 (28, 37), reflecting a moderate level, consistent with the findings of Huang et al. (2024) and Yan D. X. et al. (2021), but lower than that reported in an Egyptian study of nurse innovators (El-Gazar et al., 2024). The discrepancy in nurses’ creativity between China and Egypt may be attributed to differences in leadership style (ambidextrous vs. hierarchical), psychological safety climate, workload intensity, and innovation-oriented training. Egyptian nurses benefitted from nurse managers’ ambidextrous behaviors that enhanced psychological safety and creativity, whereas Chinese nurses often operate in a more error-averse, high-pressure environment that may inhibit creative expression. In addition, there are several reasons why nurses’ creativity has not been fully unleashed. In terms of training systems, nursing practice in China remains primarily focused on clinical care services, with most nurses lacking systematic training in courses related to nursing creativity (Yan J. J. et al., 2021). From a work perspective, the overwhelming workload disrupts the balance between work time and cognitive reflection, leaving nurses with insufficient energy for deep thinking and creativity. Furthermore, resource shortages, technical limitations, and other complex challenges in clinical practice have also hindered nurses from applying innovative thinking in their demanding clinical work to some extent, thereby affecting the translation and implementation of clinical creativity (Cheng et al., 2022).

The study findings indicate a negative correlation between nurses’ compassion fatigue and creativity (*r* = −0.698, *p* < 0.001). Regression analysis further showed that compassion fatigue directly impacts creativity (*β* = −0.593, *p* < 0.001), meaning that more severe compassion fatigue is associated with lower creativity. Prolonged exposure to patients’ illness experiences, coupled with inadequate rest and limited social support, may cause compassion strain to exceed nurses’ capacity, triggering a series of physical, emotional, and social changes (Yu et al., 2018). Consequently, compassion fatigue develops, leading to diminished work enthusiasm, reduced job commitment (Topçu et al., 2023), and heightened professional burnout (Figley, 2002; Beck, 2011). This, in turn, suppresses work motivation and impedes creative behaviors.

The results also show that nurses’ ego depletion is significantly negatively correlated with creativity (*r* = −0.656, *p* < 0.01). Higher ego depletion predicts lower creativity, consistent with the findings of Zhang et al. (2024). Ego depletion disrupts the equilibrium of nurses’ psychological resources, triggering multiple negative consequences. First, it directly leads to failure of emotional management mechanisms, accompanied by weakened professional commitment and diminished self-management behaviors. This not only reduces the effectiveness and quality of nursing care but also potentially threatens patient safety through issues such as distracted attention and impaired decision-making (Molero Jurado et al., 2021). Second, from the perspective of creativity generation, creativity requires mental agility, emotional focus, stable cognitive functions, and positive psychological states. When nurses experience ego depletion, persistent resource depletion renders them more susceptible to cognitive biases and habitual processing patterns, manifesting as underestimation of their own capabilities, lowered professional standards, and pessimistic career expectations (Fischer et al., 2007). This state further encourages a “risk-averse tendency,” leading nurses to avoid the additional challenges and trial-and-error risks required for creative work (Huang et al., 2024). Ultimately, this reduces innovative attempts and suppresses the generation and expression of creativity at the behavioral level.

The mediation analysis revealed that ego depletion partially mediated the compassion fatigue-creativity relationship (indirect effect = −0.176, 95% bootstrap CI: −0.360 to −0.027; proportion mediated = 22.89%; *p* < 0.001). Consistent with resource conservation theory (Hobfoll, 1989), compassion fatigue may lead to nurses to continuously expend emotional resources beyond normal levels (Hagger et al., 2014), leading to a cumulative resource drain that manifests as ego depletion. Once in a state of ego depletion, nurses experience impaired attentional and executive mechanisms (Ma et al., 2020), which undermines the cognitive flexibility, sustained focus, and risk-taking willingness required for creative thinking (Amabile et al., 1996). Consequently, depleted nurses tend to prioritize short-term goals that rapidly alleviate negative emotions over long-term creative efforts (Muraven and Baumeister, 2000). In summary, the partial mediation model reveals that compassion fatigue is negatively associated with creativity both directly and indirectly through ego depletion, supporting a resource-based explanation of how emotional strain impairs innovative behavior.

Chinese context specificity. The mediation model identified above should be interpreted within China’s specific healthcare and cultural context. Compared to many Western countries, Chinese nurses face more severe staffing shortages, longer working hours, and weaker psychological support systems (OECD, 2021; Aiken et al., 2014). The hierarchical “appeasement management” style common in Chinese hospitals and the Confucian norm of emotional endurance discourage nurses from seeking help or setting emotional boundaries, which is likely to accelerate the conversion of compassion fatigue into ego depletion. In contrast, nurses in individualistic Western cultures are more likely to access employee assistance programs, regular debriefing, and clinical supervision, potentially buffering the negative effects. Given the high compassion fatigue (median = 34.5) and moderately high ego depletion (median = 45.0) in our sample, the Chinese context may amplify the mediating effect size (22.89%). However, direct cross-cultural comparisons are needed to test whether this proportion is indeed larger than that in Western healthcare systems.

Practical pathways to break the vicious cycle can be derived from our quantitative results. Based on resource gain theory (Muraven and Baumeister, 2000), interventions should target three processes: reducing the initial level of compassion fatigue, attenuating the transition from compassion fatigue to ego depletion, and directly replenishing depleted psychological resources. Based on these findings, nursing managers in Chinese tertiary hospitals should implement a multi-path intervention strategy. First, to directly reduce the negative impact of compassion fatigue on creativity (Path 1), routine screening using validated tools (e.g., the Professional Quality of Life Scale) should be established, accompanied by access to psychological first aid and peer support groups. Second, to weaken the compassion fatigue–ego depletion link (Path 2), mindfulness-based stress reduction programs, emotion regulation workshops, and inclusive leadership training (including active listening, fair resource allocation, and encouraging speaking-up) are recommended (Wang et al., 2023; Wu et al., 2025; Ge et al., 2024; Tang et al., 2023). Additionally, implementing hospital-wide psychological counseling can not only significantly alleviate nurses’ disappointment, frustration, and helplessness caused by compassion fatigue, but also reduce their level of ego depletion, thereby providing direct support for the restoration of psychological resources (Chang and Chang, 2019). Third, to mitigate the detrimental effect of ego depletion on creativity (Path 3), workload-based scheduling (e.g., limiting consecutive high-acuity shifts) and protected time for creative thinking should be implemented (Chen et al., 2022). Nurses with frequent night shifts, those in high-acuity units, and those reporting poor self-rated health are high-risk groups. They should be prioritized for targeted screening and support. Fostering ambidextrous leadership among nurse managers, which balances explorative and exploitative behaviors, enhances psychological safety and encourages risk-taking even when nurses experience resource depletion. Managers who demonstrate resilience, support innovative ideas within safe boundaries, and treat mistakes as learning opportunities can mitigate the negative effect of ego depletion on creativity (El-Gazar et al., 2024).

In summary, through these multidimensional strategies, nursing managers can break the negative cycle where compassion fatigue leads to ego depletion and stifled creativity, effectively unlocking nurses’ innovative potential and enhancing their overall creativity.

## 5 Limitations

This study was conducted only in selected tertiary-level Class A hospitals in China, resulting in limitations regarding the representativeness and generalizability of the sample. Future research conducted in more diverse cultural settings would help establish the universality of these results. Although the mediation models suggest potential pathways, longitudinal studies are necessary to confirm causality and the stability of these relationships over time. Furthermore, the reliance on selfreport questionnaires introduces the possibility of social desirability bias and inaccuracies in self-perception, which could be mitigated in future studies through the use of objective measures or peer-reports. Additionally, this study did not exclude nurses with longer tenure. Resilience may increase with experience, potentially moderating the compassion fatigue–ego depletion relationship. Future studies should examine whether the mediation model differs between junior and senior nurses, as resilience might buffer the resource drain process.

## 6 Conclusion

This study provides quantitative evidence that compassion fatigue among Chinese clinical nurses directly reduces creativity and indirectly does so through ego depletion as a partial mediator. The effect sizes offer specific targets for intervention. Unlike previous research that focused on burnout or general stress, our model highlights the resource depletion chain from emotional empathic strain to self-control failure to impaired innovation.

This study demonstrates that compassion fatigue is negatively associated with creativity directly and indirectly through ego depletion (22.89% mediated). To break this negative cycle, nursing managers should implement a three-path strategy: (1) reduce compassion fatigue (e.g., via screening and psychological support), (2) weaken its transition to ego depletion (e.g., via mindfulness and inclusive leadership), and (3) buffer ego depletion’s impact on creativity (e.g., via workload scheduling and ambidextrous leadership). High-risk groups require targeted attention. These strategies offer actionable directions for nursing management.

## Data Availability

The original contributions presented in the study are included in the article/supplementary material, further inquiries can be directed to the corresponding author.
